# Influence of chronic hepatitis C infection on the monocyte-to-platelet ratio: data analysis from the National Health and Nutrition Examination Survey (2009–2016)

**DOI:** 10.1186/s12889-021-11267-w

**Published:** 2021-07-13

**Authors:** Aidan M. Nikiforuk, Mohammad Ehsanul Karim, David M. Patrick, Agatha N. Jassem

**Affiliations:** 1 School of Population and Public Health, University of British Columbia, Vancouver, British Columbia V6T 1Z4 Canada; 2 British Columbia Centre for Disease Control Public Health Laboratory, Virology, Provincial Health Services Authority, Vancouver, British Columbia V5Z 4R4 Canada; 3 Centre for Health Evaluation and Outcome Sciences, Providence Health Care, Vancouver, British Columbia V6Z 1Y6 Canada; 4 British Columbia Centre for Disease Control, Communicable Diseases and Immunization Services, Provincial Health Services Authority, Vancouver, British Columbia V5Z 4R4 Canada; 5 Department of Pathology and Laboratory Medicine, University of British Columbia, Vancouver, British Columbia V6T 1Z4 Canada

**Keywords:** Viral hepatitis, Causal inference, Machine learning, Diagnostic screening, Hepacivirus C

## Abstract

**Background:**

Hepatitis C virus (HCV) causes life-threatening chronic infections. Implementation of novel, economical or widely available screening tools can help detect unidentified cases and facilitate their linkage to care. We investigated the relationship between chronic HCV infection and a potential complete blood count biomarker (the monocyte-to-platelet ratio) in the United States.

**Methods:**

The analytic dataset was selected from cycle years 2009–2016 of the National Health and Nutrition Examination Survey. Complete case data- with no missingness- was available for *n* = 5281 observations, one-hundred and twenty-two (*n* = 122) of which were exposed to chronic HCV. The primary analysis used survey-weighted logistic regression to model the effect of chronic HCV on the monocyte-to-platelet ratio adjusting for demographic and biological confounders in a causal inference framework. Missing data and propensity score methods were respectively performed as a secondary and sensitivity analysis.

**Results:**

In the analytic dataset, outcome data was available for *n* = 5281 (*n* = 64,245,530 in the weighted sample) observations of which *n* = 122 (*n* = 1,067,882 in the weighted sample) tested nucleic acid positive for HCV. Those exposed to chronic HCV infection in the United States have 3.10 times the odds of a high monocyte-to-platelet ratio than those not exposed (OR = 3.10, [95% CI: 1.55–6.18]).

**Conclusion:**

A relationship exists between chronic HCV infection and the monocyte-to-platelet ratio in the general population of the United States. Reversing the direction of this association to predict chronic HCV infection from complete blood counts, could provide an economically feasible and universal screening tool, which would help link patients with care.

**Supplementary Information:**

The online version contains supplementary material available at 10.1186/s12889-021-11267-w.

## What is new

- *Modern techniques in causal inference were applied to understand the relationship between chronic hepatitis C virus infection and the monocyte-to-platelet ratio in complex survey data from the United States of America.*
- *Chronic hepatitis C virus infection alters a patient’s monocyte-to-platelet ratio, previous studies found an association with platelets (thrombocytopenia) but not monocytes at the population level.*
- *The monocyte-to-platelet ratio could be used as a tool to screen patients for hepatitis C virus diagnostic tests from a complete blood draw.*

## Introduction

Hepatitis C virus infects over 71 million people globally causing complex liver disease and more years of life lost than any other pathogen [1]. In 2015, the World Health Organization estimated that approximately half a million people died from HCV-related disease and three times as many were newly infected [2].

Responding to the global HCV epidemic requires country-specific strategies [3]. In the United States, fifteen to 20 % of infections progress to the chronic stage, requiring treatment [4, 5]. Undiagnosed infection can lead to liver damage, cancer and death [3]. The prognosis of HCV infection has recently improved due to the development of short course, tolerant and effective direct-acting antiviral drugs. Direct-acting antivirals clear chronic HCV infections at a 90 % rate, making the elimination of the virus plausible for the first time [3, 6]. Utilizing the full potential of direct-acting antiviral drugs for treatment-based elimination, requires the development of new tools to detect unrecognized cases and link them with care [3].

The diagnosis of chronic HCV typically involves the administration of two tests. A serological test to detect anti-HCV immunoglobulin, indicates exposure or acute infection. A positive serological result prompts the requisition of a nucleic acid amplification test (NAAT). Unlike serological methods, NAAT differentiates past exposure from acute or chronic infection by detecting viral ribonucleic acid [7]. Biomarkers offer an antigen-free alternative for the detection of a pathological process [8]. Clinical researchers have developed the aspartate aminotransferase-to-platelet ratio index (APRI) as a biomarker of late HCV infection with the purpose of predicting hepatic fibrosis [9]. We postulate that significant differences in patient biomarkers may occur early in the time course of HCV infection, enabling screening of undiagnosed chronic cases.

The described analysis examines the relationship between chronic HCV infection (exposure) and the monocyte-to-platelet ratio (MPR) (outcome) in subjects from the National Health and Nutrition Examination Survey (NHANES) cycles 2009–2016 [10]. Studies of chronic HCV have identified an association between viral load and monocyte, platelet counts [11, 12]. Immunological processes support the direction and causality of this relationship. The proliferation of HCV stimulates the innate immune system leading to an increase in the population of monocytes [13]. In a cross-sectional comparison of HCV positive- and negative- patients the percentage of monocytes in a complete blood count increased in response to infection (Δ = 0.9%, *P* < 0.001) [14]. Thrombocytopenia (a decreased platelet count) occurs in response to liver disease or interaction between the virus and the innate immune response [11]. In a previous NHANES III study, 13% of HCV seroconverted patients had a depleted platelet count while only 5% of negative patients had less than < 175 × 10^9^ platelets per litre [12]. Comparison of this result with other reports [11, 14] indicates that the expected effect size of HCV infection on complete blood counts depends on values used to categorize a normal from abnormal count. To limit misclassification bias we composed the MPR from numeric platelet and monocyte counts before categorization by the mean value of HCV negative patients. We hypothesize that complete blood counts from patients with chronic HCV will have a higher MPR than those not infected. Understanding the relationship between chronic HCV infection and MPR may allow for screening to occur from complete blood counts, making population level screening more economically feasible.

## Methods

### Data, design and study population

This cross-sectional study investigated the relationship between chronic HCV infection and MPR from complete blood counts using NHANES survey data cycles 2009 to 2016 [10]. The NHANES survey represents the non-institutionalized civilian population of fifty American states and the district of Columbia through complex sampling design. The design includes primary sampling units, strata and sample weights, a full description of how these measures were derived is available from the Centers for Disease Control and Prevention [15]. Oversampling was conducted for those greater than 80 years of age and identifying as an African American, Asian or Hispanic ethnicity. Survey features were included in the analysis to generalize the findings to the population of the United States [16, 17]. The aggregated medical examination center weights were divided by the duration of the study in two-year survey cycles (*n* = 4) (Additional file 1: Table S1). The University of British Columbia’s Policy 89, item 7.10.3 on studies involving human participants and Article 2.2 of the Tri-Council Policy Statement on Ethical Conduct for Research Involving Humans, provide ethical support of the study [18, 19]. NHANES is a publicly available dataset administered by the National Center for Health Statistics, written informed consent was provided by all participants [20].

### Analytic sample and variable selection

In the analytic dataset, we have included the exposure (chronic HCV infection) and outcome (monocyte-to-platelet ratio) of interest, select covariates or potential confounders in the relationship between chronic HCV infection and MPR. The exposure of interest (LBXHCR) categorically measures chronic HCV infection by NAAT where: “1” signals positive, “2”- negative, “3”- no seroconversion (lack of HCV specific antibodies) and “.” as missing (Additional file 1: Table S1) [15]. Exposure was releveled to “1” as positive and “0” as negative or no seroconversion. The outcome variable (MPR) was constructed from complete blood count measures of monocyte count (“LBDMONO”) and platelet count (“LBXPLTSI”) (Additional file 1: Table S1) [15]. The monocyte-to-platelet ratio (MPR) was calculated by dividing thousands of monocytes per microliter by thousands of platelets per microliter. The liquid volume measurement was then converted to milliliters. All variables in the analytic dataset were renamed, coded or leveled to ease interpretation (Additional file 1: Table S1).

**Fig. 1:**
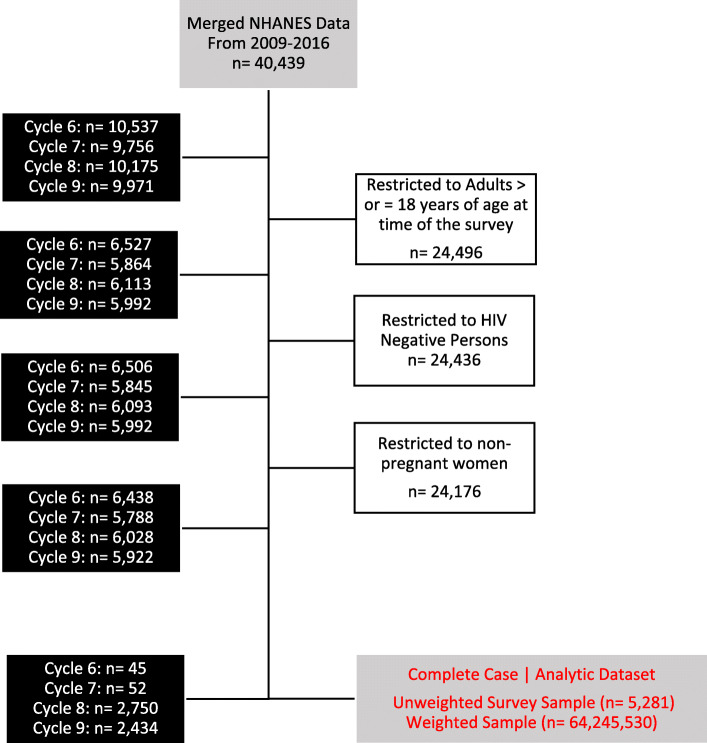
Illustration of the exclusion criteria applied to generate an analytic dataset from a merger of national health and nutrition examination survey cycle years 2009 to 2016. The described exclusion criteria were applied for the following reasons: HIV infection depletes the innate immune system and may affect monocyte count; pregnancy was considered to affect female complete blood cell counts and the dataset was restricted to subjects ≥ 18 years of age as complete blood cell counts differ by developmental stage. Cycle refers to the two-year NHANES data cycle (SDDSRVYR) from which the data was retrieved. Following application of the exclusion criteria, *n* = 18,895 observations were omitted as they lacked complete case information across 15 variables. The complete case analytic dataset contains *n* = 5281 unweighted observations from the survey sample, one-hundred and twenty-two (*n* = 122) of which were exposed to chronic HCV

Covariates were identified from previous population level studies of HCV patients and plotted in a directed acyclic graph (DAG) (Additional file 1: Figure S1) [21, 22]. The selected covariates portray measurements of general health status, social-economic status, biological sex, age, race, marital status, transfusion status, white blood cell count, needle use, average alcohol intake per day, anemia, cancer or diabetes diagnosis (Additional file 1: Figure S1). Exclusion criteria removed subjects with a positive human immunodeficiency virus test result, those pregnant or less than 18 years of age (Fig. 1). After excluding *n* = 18,895 observations for missing information on fifteen variables, *n* = 5281 individuals with complete case data were identified, of whom *n* = 112 had chronic HCV infection (Table 1, Figure S1). Confounders were selected by the disjunctive cause criterion and included throughout automated variable selection (Additional file 1: Figures S1, S2) [18]. Covariates which did not meet the requirements of the disjunctive cause criterion were selected for inclusion by automated variable selection; therefore, they were only included if they increased the model fit and precision of the estimates.

**Table 1:** Structure and characteristics of the complete case dataset from the National Health and Nutrition Examination Survey study period, 2009–2006 to investigate the effect of chronic hepatitis C virus infection on the monocyte-to-platelet ratio

Variable Name	Level	Total	Low MPR	High MPR	Rao-Scott X^2^a^
	.	5281 [64,245,530]^b^	2754 (0.54)	2527 (0.45)	
**Chronic HCV = Positive (%)**	.	122	42 (0.90)	80 (2.5)	< 0.001
**Age** (mean [SD])	.	.	42.74 (13.64)	43.93 (14.36)	0.054
**Sex** = Male	.	2759	1220 (40.3)	1539 (65.1)	< 0.001
**Race** (%)	.				< 0.001
	Black	1079	626 (10.9)	453 (9.0)	
	White	2127	1091 (65.4)	1036 (71.3)	
	Hispanic	1377	797 (15.4)	580 (13.5)	
	Other	698	455 (8.30)	243 (6.20)	
**General Health** (%)	.				0.022
	Excellent	462	277 (10.80)	185 (8.80)	
	Very Good	1487	877 (35.30)	610 (32.30)	
	Good	3188	1747 (52.40)	1441 (56.60)	
	Poor	144	68 (1.50)	76 (2.30)	
**Transfusion** = Yes (%)	.	427	242 (7.80)	185 (8.3)	0.723
**Needle Use** = Yes (%)	.	146	63 (2.60)	83 (3.10)	0.479
**Married** = Lives Alone (%)	.	2094	1202 (36.0)	892 (34.50)	0.312
**Social Economic Status** (%)	.				0.88
	≤ 1.30	1650	887 (20.70)	763 (21.80)	
	≤ 1.85	699	396 (11.10)	303 (10.80)	
	> 1.85	2824	1624 (66.3)	1200 (65.5)	
**White Blood Cell Count** (mean [SD])	.	.	6.81 (1.93)	8.00 (2.34)	< 0.001
**Cancer Diagnosis** = Yes (%)	.	311	186 (8.70)	125 (6.90)	0.028
**Anemia** = Yes (%)	.	515	117 (3.70)	62 (2.10)	0.006
**Diabetes Diagnosis** (%)					0.003
	Not Diabetic	4663	2672 (92.2)	1991 (88.6)	
	Pre-Diabetic	103	49 (1.40)	54 (2.30)	
	Diabetic	515	248 (6.40)	267 (9.10)	
**Average Alcohol Consumption per Day** (mean [SD])	.	.	3.30 (29.13)	3.54 (15.22)	0.789

^a^ The Rao-Scott Chi Square test was used to test for independence between each of the described covariates and monocyte-to-platelet ratio in survey weighted data (α = 0.05)

Percentages represent the unweighted population of the NHANES study base

^b^ the weighted survey sample number of participants with complete case data in the study

### Statistical analysis

#### Transformation of the monocyte-to-platelet ratio

The monocyte-to-platelet ratio was dichotomized by linear regression [9]. As described by the theory of mean-of-class classification, linear regression was used to find the mean MPR of HCV negative participants in the complete case dataset [23, 24]. The continuous numeric MPR variable was then dichotomized to a factor with two levels. Logistic regression and a receiver operator characteristic curve analysis were used to predict HCV infection by dichotomized MPR for transparency, and to show that we did not choose a biased value for categorization.

#### Descriptive statistics

Characteristics of the analytic sample were determined by unweighted survey-sample number and as a weighted sample percentage, representing the population percentage. The sample was stratified by the outcome of interest (MPR), independence was tested between the outcome and covariates via the Rao-Scott Chi-square test for survey design [25].

#### Statistical inference

Primary analysis used survey-weighted logistic regression to model the relationship between chronic HCV and MPR adjusting for known confounders (i.e. age, type 2 diabetes mellitus and needle use) [11, 26–28]. Covariates were chosen through literature review, confounders were selected by the disjunctive cause criterion and use of a directed acyclic graph (DAG) [29, 30]. Missing data and propensity score analyses were performed secondarily and to assess sensitivity of the results.

The primary analysis utilized a survey-weighted logistic regression model to estimate the effect of chronic HCV infection on MPR [17]. Confounders defined by use of a DAG were included in the model. Covariates selected by literature review which did not meet the definition of a confounder were selected for inclusion in the model by backward elimination on the Akaike information criterion (AIC) [31]. Identified confounders were locked in the backward elimination algorithm, so that only covariates which did not meet the definition of a confounder were evaluated for inclusion by automated variable selection (Additional file 1: Figure S2). Collinearity was assessed by the variance inflation factor, with a cutoff of 5 [32]. The primary analysis was performed in the complete case dataset, using the R packages: tidyverse, dataexplorer, jtools, car, tableone, survey, publish, MASS, pROC and caret.

The secondary analysis used multiple imputation by chained equations to provide missing data on the covariate needle use. Under the assumption of missing at random, predictors of needle use were selected by a minimum correlation of 5 % [33]. Missing data were imputed in ten datasets with predictive mean matching, the number of iterations was set to match the percentage missing (12.87). A survey-weighted logistic regression model was fit to each imputed dataset to estimate the effect of chronic HCV infection on MPR. The resulting odds ratios were pooled by Rubin’s rules [33]. A secondary analysis was performed in the imputed dataset, using the R packages: mice and mitools.

#### Sensitivity analysis

The sensitivity of the primary and secondary analysis was evaluated by weighted propensity score analysis [34]. The propensity scores were generated by tenfold cross-validated ensemble machine learning. The super learner was specified to predict by: a general linear model, elastic net, bootstrap aggregation and classification, regression training [35]. The ensembled propensity scores were applied by inverse proportional weighting to estimate the average treatment effect [36]. Weights were scaled by the mean and truncated by the first and ninety-ninetieth percentile. Changes in covariate balance after propensity score weighting was measured by reduction of standardized mean difference (SMD) [36]. Covariates unbalanced by propensity score weighting (SMD > 0.2) were adjusted for in the outcome model. Sensitivity analysis was performed in the complete case dataset, using the R packages: superlearner and cobalt. All statistical analysis was conducted in R version 3.6.1 [37].

## Results

### Statistical analysis

#### Transformation of the monocyte-to-platelet ratio (MPR)

The MPR was calculated from the NHANES variables “LBDMONO” and “LBXPLTSI” and converted to 1000 cells/ml. In the analytic dataset, the value of MPR ranges from 0.27–22.22 with a mean of 2.53 and standard deviation of 1.05. Linear regression found a positive association between chronic HCV infection and MPR. MPR was categorized with a cut point of 2.49, Low (min-2.49), High (> 2.49-max). Logistic regression was used to evaluate the accuracy of this cut point in combination with a receiver operator characteristic curve analysis (sensitivity of 65% and a specificity of 54%) (Additional file 1: Figure S3).

#### Descriptive statistics

The analytic dataset includes *n* = 5281 (*n* = 64,245,530 after weighting the sample) observations with *n* = 122 (*n* = 1,067,882 sample weighted) exposures to chronic HCV infection, the outcome was recorded for all exposures (Table 1). Bivariate analysis of the relationship between chronic HCV infection and MPR found a positive correlation, 66% of persons with chronic HCV infection have a high MPR (> 2.49) (*P* < 0.001) (Table 1). Characteristics of the analytic sample were determined by unweighted survey-sample number and as a weighted sample percentage, representing the population percentage (Table 1).

### Statistical inference

#### Primary analysis

A survey-weighted logistic regression model was built by use of a DAG and AIC backward elimination to estimate the effect of chronic HCV infection on MPR with adjustment of potential confounders (Table 2). Notably, none of the covariates which did not meet the definition of a confounder were found to increase model fit or precision of the effect estimates during automated variable selection. All the confounders illustrated in the DAG were included throughout automated variable selection. The variables chronic HCV infection, age, sex, race, white blood cell count (WBC) and cancer diagnosis exhibit a statistically significant relationship with MPR (*P* < 0.05). No variables were removed from the parsimonious model selected by Akaike information criterion. No collinearity was detected with race having the greatest VIF value equal to 3.77. The model selected for primary analysis (Table 2) estimates that those chronically infected with HCV have 3.10 times the odds of a high MPR score than those not exposed (OR = 3.10, [95%CI: 1.55–6.18]).

**Table 2:** Results of survey weighted logistic regression analysis in the relationship between chronic hepatitis C infection and categorized monocyte-to-platelet ratio (low, high): from a complete case dataset of the National Health and Nutrition Examination Survey, 2009–2016

Variables	Reference	Level	Adjusted Odds Ratio	Confidence Interval^a^
**Chronic HCV**	Negative	.		
	.	Positive	3.10	1.55–6.18
**Age**	.	.	1.01	1.00–1.02
**Race**	Black	.		
	.	White	1.12	0.93–1.36
	.	Hispanic	0.78	0.62–0.98
	.	Other	0.78	0.60–1.01
**Sex**	Female	.		
	.	Male	3.35	2.89–3.88
**Needle Use**	No	.		
	.	Yes	0.63	0.35–1.13
**Diabetes**	Not Diabetic	.		
	.	Pre-Diabetic	1.77	0.90–3.47
	.	Diabetic	1.06	0.79–1.43
**Cancer**	No Diagnosis	.		
	.	Diagnosis	0.74	0.56–0.99
**WBC**	.	.	1.39	1.33–1.45

^a^ the 95% confidence interval for the given estimate

Multi-collinearity was tested by the variable inflation factor, no inflation was detected < 5

#### Secondary analysis

**Fig. 2:**
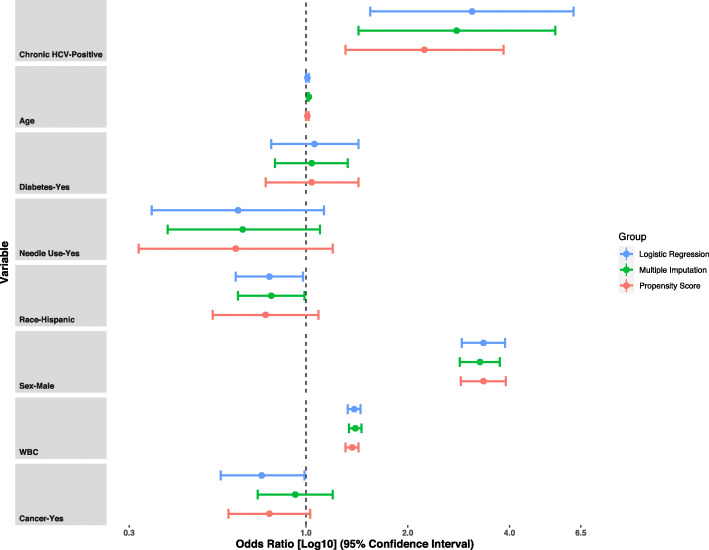
Results of survey weighted analysis examining the relationship between chronic Hepatitis C infection and monocyte-to-platelet ratio in complete case and missing data from the National Health and Nutrition Examination Survey cycle years 2009 to 2016. The adjusted odds ratio and 95% confidence intervals shown in the log scale for statistically significant covariates in the primary analysis, secondary analysis or those requiring adjustment after propensity score weighting as a sensitivity analysis. All analyses incorporated survey features from the medical examination center derived weight following the methodology of Ridgeway et al. [34]

The association between exposure to chronic HCV infection and MPR was also investigated by multiple imputation of the covariate needle use. The minimum correlation matrix selected: age, transfusion status, sex, monocyte count, platelet count, cancer, and diabetes as predictors of needle use. A total of seven hundred and sixty-eight observations (*n* = 768) were added by multiple imputation, including thirteen additional exposures (*n* = 13). The outcome (MPR) was recorded for all the imputed data. Combined estimates from the ten imputed datasets show that those chronically infected with HCV have 2.79 times the odds of a high MPR score than those not exposed (OR = 2.79, [95%CI: 1.43–5.46]) (Fig. 2).

### Sensitivity analysis

The sensitivity of the primary and secondary analysis was tested by propensity score weighting. Propensity scores were predicted for HCV exposure by ensemble machine learning. The standardized mean differences of the covariates: age, race, sex, general health status, transfusion status, needle use, marital status, social-economic status did not meet the < 0.2 cut-off; therefore, they were adjusted for in the outcome model. Propensity score weighting estimated that those chronically infected with HCV have 2.24 times the odds of a high MPR score than those not exposed (OR = 2.24, [95%CI: 1.31–3.84]) (Fig. 2).

We do not interpret effect estimates for variables other than the primary exposure as doing so, may require specification of a separate causal framework to account for additional confounding [38].

## Discussion

### Summary of results

The primary analysis found a strong measure of association between chronic HCV infection and MPR in the population of the United States, after adjusting for the confounders: age, race, sex, needle use, diabetes, white blood cell count and cancer. Americans with chronic HCV infection have 3.10 times or 210% higher odds of a high MPR score, than those not exposed. The adjusted effects of age, sex and needle use agree with known literature, exposure to injectable drugs was found to decrease monocyte count in a mouse model and within a cohort of opioid users [39, 40]. The secondary and sensitivity analysis agreed with the primary results, suggesting that MPR differs in those chronically infected and not infected with HCV.

### Comparison with literature

Past studies describe an association between chronic HCV infection and low platelet count [11, 41]. The mechanism of this relationship remains poorly understood. The virus may trigger auto-immunogenicity and decrease the production of thrombopoietin [42].

Chronic HCV infection affects monocyte count through stimulation of the innate and adaptive immune response [13]. Monocytes differentiate into dendritic cells or macrophage in the human immune system. HCV infects both cell types, which serve as reservoirs for viral replication in vivo [43]*.* Conceptually, monocyte count may indicate the presence and severity of HCV infection.

Tsai et al. [14] analyzed the effect of chronic HCV infection on blood cell counts adjusting for the demographic confounders of age and sex. They found that monocyte count positively, and platelet count negatively associated with HCV viral load. Monocyte count increased by one-thousand cells per microliter in patients who seroconverted.

Streiff et al. [12] conducted an NHANES study to understand peripheral blood count abnormalities in patients exposed to HCV. An association was found between HCV exposure and platelet counts at cutoff (OR = 2.33, [95% CI: 1.08–5.02]; no relationship was found for monocyte count.

In summary, monocyte count increases and platelet count decreases in response to chronic HCV infection, as the odds of a high MPR were greater in those exposed. The population-level differences in MPR between those infected and not infected with HCV in the United States make it a candidate screening tool for chronic HCV infection. Performance of the MPR as a screening tool depends on its predictive accuracy (sensitivity/ specificity), the prevalence of HCV in the target population and availability of other tests in the resource setting. Theoretically, implementation of the MPR as a screening tool for chronic HCV infection may have the most impact in a low-resource, high-prevalence setting where clinicians could leverage previously reported complete-blood-count data before follow-up with a secondary screening test or a confirmatory NAAT type diagnostic.

### Strengths and limitations

The presented study possesses strength in design, analysis, and interpretability. The single temporal collection point of cross-sectional studies evokes the issue of reverse causality [44]. In the NHANES sample, variable characteristics were collected by means of an interview or through the medical examination center (MEC) [15]. The natural history of HCV infection establishes temporal causality in the relationship with complete blood cell counts. The MEC tested for HCV-RNA by NAAT and the incubation period of HCV lasts between 2 to 26 weeks with an average of 7 weeks [45]. Therefore, if a person tests HCV positive at the MEC they were initially exposed to the virus at least 2 weeks before, establishing unidirectional causality.

Two additional analyses were conducted (multiple imputation and propensity score weighting) both support the robustness of a positive relationship between chronic HCV infection and MPR. The variable needle use was imputed because, it meets the criterion of missing at random [33]. Persons who inject drugs are less likely to report doing so to a federal agency than to report no needle use [46]. Therefore, imputation served to help estimate a more accurate rate of needle use in the population of the United States. In addition to reducing bias, missing data analysis also aids precision and increases statistical power [33]. Weighted propensity score analysis allows estimation of the average treatment effect (ATE), a measure commonly deployed in randomized clinical trials where the control and treatment groups exhibit exchangeability. In cross-sectional studies, ATE allows estimation of treatment effect when the observed groups have a similar probability of exposure to the selected confounders [47]. In studies on biomarkers, confounding by indication due to the effect of a drug prescribed for a comorbidity can alter the distribution of a marker in those treated [48]. Propensity score weighting partially accommodates for this bias, as the comorbidity serves in the surrogate of confounding from the drug. The exposed and non-exposed groups have a more equal probability of exposure to the drug through the similar probability of exposure to the indication [49]. The use of NHANES data across several collection years benefits the ability to generalize from and the validity of our study. Past analysis of NHANES data has found true population-level effects such as the role of high cholesterol in heart disease [15].

Weaknesses of the study include the potential for unmeasured confounding, such as co-infection. Risk factors for HCV exposure overlap with those of other chronic infectious diseases like hepatitis B and tuberculosis [50]. Laboratory data confirming active infection with these pathogens was not publicly available in the NHANES dataset [15]. The variable white blood cell count (WBC) was selected as a measure of proxy confounding, with the conceptual understanding that exposure to a foreign antigen increases WBC [51]. The possibility also exists that co-infection acts as an independent predictor or mediates the effect of chronic HCV infection on MPR [52]. Due to the cross-sectional study design, it was not possible to evaluate how the duration of HCV infection affects MPR. In the age of interferon-based therapy, Ikeda et al. [53] found that low platelet count, before the initiation of treatment, was a significant risk factor in the development of hepatocellular carcinoma (HCC) during follow-up. The ability of low platelet count to predict late-stage liver pathology in those with HCV infection means that thrombocytopenia may occur earlier in the disease process than liver fibrosis or HCC [53].

### Clinical and epidemiological interpretation

Analysis by logistic regression adjusted for the confounding variables: age, race, biological sex, needle use, white blood cell count, cancer diagnosis, and diabetes all share a dependent relationship with MPR. The relationship between these confounders and platelet count is unexplainable because, expected platelet counts are not well described for healthy populations [14, 54]. The cut point to categorize MPR in our study can be interpreted in the range of normal complete blood counts in the United States. The division of the lower normal limit of expected monocyte count by platelet count produces a MPR of approximately 1.33 [55]. We caution overinterpretation of this value as true population-level counts of monocytes and platelets are not well understood and can vary. An MPR of 1.33 could be derived from counts below or above the normal range.

### Future directions

Future analysis should focus on investigating the relationship between chronic HCV infection and MPR in a country other than the United States. Geographical validation would strengthen the conclusion that chronic HCV infection affects MPR. Unmeasured confounding should also be assessed by adjustment for relevant co-infections and prescription of immune-modulating drugs. A logical follow up to this work would be the use of the MPR alone or in combination with other risk factors to build a predictive model to screen for chronic HCV infection. We caution that a biomarker like the MPR should not be used independently for diagnosis of HCV infection but in combination with a specific serological or NAAT type test. Implementation of the MPR in a reflex testing algorithm where a “high” score would requisition a follow up test could benefit the positive predictive value of diagnosis, conserve tests in resource limited settings and make population level screening initiatives economically viable. The primary benefit of population level screening with the MPR being that it would enhance surveillance for HCV infection. Persons that may not otherwise be tested for HCV, could be identified from a complete blood count. Subsequent research efforts should test the diagnostic accuracy of the MPR in a study population, or work to identify additional complete blood count-based measurements- which are associated with chronic HCV infection- and may increase the discrimination of MPR as a predictive tool.

## Conclusion

A relationship exists between chronic HCV infection and the complete blood count biomarker (monocyte-to-platelet ratio) in the population of the United States. We conclude that generally chronic HCV infection increases monocyte count and decreases platelet count. Understanding the relationship between chronic HCV and MPR, could advise a biomarker-based screening tool for chronic HCV infection, similar to the use of the APRI for liver fibrosis [9]. Development of an antigen free, biomarker-based, screening tool for chronic HCV infection would have tremendous economic benefits and decrease the time-to-treatment through the initiation of care from a complete blood draw.

## Supplementary Information

**Additional file 1: Figure S1.** Directed acyclic graph of variables considered in building an effect size model of the relationship between chronic hepatitis C infection and the monocyte-to-platelet-ratio from the National Health and Nutrition Examination Survey, 2009–2016. **Figure S2.** Logistic regression model building using a directed acyclic graph for definition of confounders and automated variable selection by backward elimination for undefined covariates. **Figure S3.** Receiver operating characteristic curve for threshold analysis of the monocyte-to-platelet-ratio as a lone predictor of chronic hepatitis C infection in analytic data from the National Health and Nutrition Examination Survey, 2009–2016. **Table S1.** Description of variables involved in analysis of the relationship between chronic hepatitis C infection and the monocyte-to-platelet ratio from the National Health and Nutrition Examination Survey, 2009–2016.

## Data Availability

The datasets analyzed in the described study are available as part of the NHANES repository, https://wwwn.cdc.gov/nchs/nhanes/Default.aspx.
