# Supplementary material for: Influence of chronic hepatitis C infection on the monocyte-to-platelet ratio: data analysis from the National Health and Nutrition Examination Survey (2009–2016)
Source: BMC Public Health. 2021 Jul 13;21:1388. doi: 10.1186/s12889-021-11267-w (PMC8278694; doi:10.1186/s12889-021-11267-w)
Supplement: Supplementary file 1 — Additional file 1: Figure S1. Directed acyclic graph of variables considered in building an effect size model of the relationship between chronic hepatitis C infection and the monocyte-to-platelet-ratio from the National Health and Nutrition Examination Survey, 2009–2016. Figure S2. Logistic regression model building using a directed acyclic graph for definition of confounders and automated variable selection by backward elimination for undefined covariates. Figure S3. Receiver operating characteristic curve for threshold analysis of the monocyte-to-platelet-ratio as a lone predictor of chronic hepatitis C infection in analytic data from the National Health and Nutrition Examination Survey, 2009–2016. Table S1. Description of variables involved in analysis of the relationship between chronic hepatitis C infection and the monocyte-to-platelet ratio from the National Health and Nutrition Examination Survey, 2009–2016. [file 12889_2021_11267_MOESM1_ESM.docx]

Title: Influence of Chronic Hepatitis C Infection on the Monocyte-to-Platelet Ratio: Data Analysis from the National Health and Nutrition Examination Survey (2009-2016)

Running Title: Influence of Chronic Hepatitis C Infection on the Monocyte-to-Platelet Ratio

Aidan M. Nikiforuk^1,5^, Mohammad E. Karim^1,2^, David M. Patrick^1,4^, Agatha N. Jassem^3,5^

^1^ School of Population and Public Health, University of British Columbia, Vancouver, British

Columbia, Canada, V6T 1Z4.

^2^ Centre for Health Evaluation and Outcome Sciences, Providence Health Care, Vancouver,

British Columbia, Canada, V6Z 1Y6.

^3^ Department of Pathology and Laboratory Medicine, University of British Columbia, Vancouver,

British Columbia, Canada, V6T 1Z4.

^4^ British Columbia Centre for Disease Control, Communicable Diseases and Immunization

Services, Provincial Health Services Authority, Vancouver, British Columbia, Canada, V5Z 4R4.

^5^ British Columbia Centre for Disease Control Public Health Laboratory, Virology, Provincial

Health Services Authority, Vancouver, British Columbia, Canada, V5Z 4R4.

**KEY WORDS**Viral Hepatitis, Causal Inference, Machine Learning, Diagnostic Screening, Hepacivirus C

**Corresponding Author:** Dr. Agatha Jassem, Department of Pathology and Laboratory Medicine, University of British Columbia, and British Columbia Centre for Disease Control Public Health Laboratory. Email: agatha.jassem@bccdc.ca.

**SUPPLEMENTARY MATERIALS:**

**Figure S1:** Directed acyclic graph of variables considered in building an effect size model of the relationship between chronic hepatitis C infection and the monocyte-to-platelet-ratio from the National Health and Nutrition Examination Survey, 2009-2016.

Variables were renamed and re-leveled for the study from the National Health and Nutrition Examination Survey (Table S1). An effect size model was built to measure the direct effect of HCV nucleic acid amplification test result on the monocyte-to-platelet ratio by application of the modified disjunctive cause criterion. Variable in green: Alcohol Consumption, General Health Status, Transfusion and Social Economic Status were omitted as they are either instrument or collider class and/or did not increase precision of the model as measured by AIC backward elimination. Variables in blue: Anemia was considered as an independent predictor and omitted from the model by maximum likelihood estimation. Variables in white: Age, Cancer Diagnosis, Diabetes, Needle Use, Race, Sex, White Blood Cell Count were considered confounders and adjusted for in the model. Variables in grey: Syndemic Co-Infection and Pharmaceutical Prescription were considered as unmeasured confounders and partially adjusted for by propensity score modelling.

**Figure S2:** Logistic regression model building using a directed acyclic graph for definition of confounders and automated variable selection by backward elimination for undefined covariates.

A parsimonious logistic regression model was built for primary analysis in the analytic dataset by a manual and automated variable selection process. In manual variable selection, a directed acyclic graph and the modified disjunctive cause criterion were used to select confounders (black) in the relationship between chronic HCV infection and monocyte-to-platelet ratio. Variables defined as confounders were locked into the model for automated selection. Only covariates which did not meet the definition of a confounder were evaluated for inclusion during automated variable selection. None of the covariates were found to increase the fit or precision of the model based on backward elimination via AIC; therefore, only defined confounders were included in the parsimonious model.

**Figure S3:** Receiver operating characteristic curve for threshold analysis of the monocyte-to-platelet-ratio as a lone predictor of chronic hepatitis C infection in analytic data from the National Health and Nutrition Examination Survey, 2009-2016.

A threshold of 2.49 was selected to categorize the Monocyte-to-Platelet Ratio (MPR) as Low (min- 2.49) and High (>2.49-max), with a sensitivity of 65% and specificity of 54%. The sensitivity or specificity of the MPR to predict chronic HCV infection could be improved with the addition of additional explanatory variables to the classification model. We did not include further variables in this analysis as our priority was to adjust for confounding control in the relationship between chronic HCV infection (exposure) and MPR (outcome).

**Table S1:** Description of variables involved in analysis of the relationship between chronic hepatitis C infection and the monocyte-to-platelet ratio from the National Health and Nutrition Examination Survey, 2009-2016.

| Variable Name in NHANES | Levels in NHANES | Variable Name in Analytic Data | Levels in Analytic Data | Class | Relationship | Description* |
| --- | --- | --- | --- | --- | --- | --- |
| WTMEC2YR | Continuous | Full Sample 2 Year Weights | Continuous | Numeric | Survey Feature | Weight statement to account for the unequal probability of sampling, survey nonresponse and adjustments to population control totals |
| SDMVPSU | Continuous | Masked variance pseudo-PSU | Continuous | Numeric | Survey Feature | Cluster statement to specify primary sampling unit (sdmvpsu) — this accounts for the design effects of clustering |
| SDMVSTRA | Continuous | Masked variance pseudo-stratum | Continuous | Numeric | Survey Feature | Stratum statement to specify the strata (sdmvstra) -- this accounts for the design effects of stratification |
| MCQ220 | Yes  No  Refused Don't know  Missing | Cancer Diagnosis | No cancer diagnosis  Diagnosed with cancer | Categorical | Confounder | {Have you/Has SP} ever been told by a doctor or other health professional that {you/s/he} had cancer or a malignancy (ma-lig-nan-see) of any kind? |
| ALQ130 | Continuous | Average Alcohol Consumption per Day | Continuous | Numeric | Covariate | In the past 12 months, on those days that {you/SP} drank alcoholic beverages, on the average, how many drinks did {you/he/she} have? |
| DIQ010 | Yes  No  Borderline Refused Don't know  Missing | Diabetes Diagnosis | Not diabetic  Diabetic Pre-diabetic | Categorical | Confounder | The next questions are about specific medical conditions. {Other than during pregnancy, {have you/has SP}/{Have you/Has SP}} ever been told by a doctor or health professional that {you have/{he/she/SP} has} diabetes or sugar diabetes? |
| MCQ053 | Yes  No  Refused  Don't know  Missing | Anemia Diagnosis | Yes  No | Categorical | Covariate | During the past 3 months, {have you/has SP} been on treatment for anemia (a-nee-me-a), sometimes called "tired blood" or "low blood"? [Include diet, iron pills, iron shots, transfusions as treatment.] |
| LBXWBCSI | Continuous | White Blood Cell Count | Continuous | Numeric | Confounder |  |
| RIDEXPRG | Yes, positive lab pregnancy test or self-reported pregnant at exam The participant was not pregnant at exam Cannot ascertain if the participant is pregnant at exam missing | Pregnant | Pregnant Not pregnant | Categorical | Exclusion | Pregnancy status for females between 20 and 44 years of age at the time of MEC exam. |
| DMDMARTL | Married Widowed Divorced Separated Never married Living with partner Refused Don't know Missing | Marital Status | Lives with partner Lives alone | Categorical | Covariate | Marital status |
| LBDMONO | Continuous | Monocyte Count | Continuous | Numeric | Outcome | Monocyte number (1000 cells/uL) |
| LBXPLTSI | Continuous | Platelet Count | Continuous | Numeric | Outcome | Platelet count (1000 cells/uL) |
| LBXHCR | Positive  Negative Negative Screening HCV Antibody Missing | HCV NAAT/ Chronic HCV | Negative Positive | Categorical | Exposure | Hepatitis C RNA |
| LBXHIVC | HIV-1/2 Reactive HIV-1/2 Non-reactive Missing | HIV | HIV-1/2 Reactive HIV-1/2 Non-reactive | Categorical | Exclusion | HIV-1, 2 Combo Serological Test |
| RIDRETH1 | Mexican American Other Hispanic Non-Hispanic White Non-Hispanic Black Other race- inc multi race Missing | Race | Black White Hispanic Other | Categorical | Confounder | Recode of reported race and Hispanic origin information |
| RIDAGEYR | Continuous | Age | Continuous | Numeric | Confounder | Age in years of the participant at the time of screening. Individuals 80 and over are top coded at 80 years of age. |
| RIAGENDR | Male Female Missing | Sex | Male Female | Categorical | Confounder | Gender of the participant. |
| INDFMMPC | Monthly poverty level index <= 1.30  1.30 < Monthly poverty level index <= 1.85  Monthly poverty level index > 1.85  Refused Don't know Missing | Social Economic Status | <= 1.30   <= 1.85   > 1.85 | Categorical |  | Family monthly poverty level index categories. |
| HSD010 | Excellent Very Good Good Fair Poor? Refused Don't know Missing | Health Status | Excellent Good Very Good Poor | Categorical | Covariate | Next I have some general questions about {your/SP's} health. Would you say {your/SP's} health in general is . . . |
| MCQ092 | Yes No Refused Don't know Missing | Transfusion | Yes No | Categorical | Covariate | {Have you/Has SP} ever received a blood transfusion? |
| DUQ370 | Yes No Refused Don't know Missing | Needle Use | Yes No | Categorical | Confounder | The following questions are about the different ways that certain drugs can be used. Have you ever, even once, used a needle to inject a drug not prescribed by a doctor? |

*Descriptions are copied directly from the National Health and Nutrition survey documentation (13).
